# A state-wide implementation of a whole of hospital sepsis pathway with a mortality based cost-effectiveness analysis from a healthcare sector perspective

**DOI:** 10.1371/journal.pgph.0000687

**Published:** 2023-05-19

**Authors:** Natasha K. Brusco, Kelly Sykes, Allen C. Cheng, Camilla Radia-George, Douglas Travis, Natalie Sullivan, Tammy Dinh, Sarah Foster, Karin Thursky, on behalf of the Safer Care Victoria “Think sepsis. Act fast” Scaling Collaboration

**Affiliations:** 1 Rehabilitation, Ageing and Independent Living (RAIL) Research Centre, Monash University, Frankston, Australia; 2 College of Science, Health and Engineering, La Trobe University, Bundoora, Australia; 3 Health Economics Department, Alpha Crucis Group, Langwarrin, Australia; 4 Department of Health, Victoria State Government, Melbourne, Australia; 5 Monash Infectious Diseases, Monash Health and School of Clinical Sciences, Monash University, Clayton, Australia; 6 School of Public Health and Preventive Medicine, Monash University, Clayton, Australia; 7 Department of Infectious Diseases, Alfred Health, Melbourne, Australia; 8 EACH, Ringwood, Australia; 9 Department of Medicine, Western Health, Melbourne, Australia; 10 Department of Infection Control, Peninsula Health, Frankston, Australia; 11 Guidance Group, Royal Melbourne Hospital, Melbourne, Australia; 12 Department of Infectious Diseases, National Centre for Antimicrobial Stewardship, Melbourne Medical School, University of Melbourne, Melbourne, Australia; 13 Department of Health Services Research, Deputy Head of Infectious Diseases, Peter MacCallum Cancer Centre, Melbourne, Australia; McGill University, CANADA

## Abstract

With global estimates of 15 million cases of sepsis annually, together with a 24% in-hospital mortality rate, this condition comes at a high cost to both the patient and to the health services delivering care. This translational research determined the cost-effectiveness of state-wide implementation of a whole of hospital Sepsis Pathway in reducing mortality and/or hospital admission costs from a healthcare sector perspective, and report the cost of implementation over 12-months. A non-randomised stepped wedge cluster implementation study design was used to implement an existing Sepsis Pathway (“Think sepsis. Act fast”) across 10 of Victoria’s public health services, comprising 23 hospitals, which provide hospital care to 63% of the State’s population, or 15% of the Australian population. The pathway utilised a nurse led model with early warning and severity criteria, and actions to be initiated within 60 minutes of sepsis recognition. Pathway elements included oxygen administration; blood cultures (x2); venous blood lactate; fluid resuscitation; intravenous antibiotics, and increased monitoring. At baseline there were 876 participants (392 female (44.7%), mean 68.4 years); and during the intervention, there were 1,476 participants (684 female (46.3%), mean 66.8 years). Mortality significantly reduced from 11.4% (100/876) at baseline to 5.8% (85/1,476) during implementation (p>0.001). Respectively, at baseline and intervention the average length of stay was 9.1 (SD 10.3) and 6.2 (SD 7.9) days, and cost was $AUD22,107 (SD $26,937) and $14,203 (SD $17,611) per patient, with a significant 2.9 day reduction in length of stay (-2.9; 95%CI -3.7 to -2.2, p<0.01) and $7,904 reduction in cost (-$7,904; 95%CI -$9,707 to -$6,100, p<0.01). The Sepsis Pathway was a dominant cost-effective intervention due to reduced cost and reduced mortality. Cost of implementation was $1,845,230. In conclusion, a well-resourced state-wide Sepsis Pathway implementation initiative can save lives and dramatically reduce the health service cost per admission.

## Introduction

With global estimates of 15 million cases of sepsis annually [1], this condition comes at a high cost to both the patient and to the health services delivering care. Mortality within 30-days is estimated at 24% [2], with those who survive the acute period experiencing an additional one-year mortality of 16% [3]. While the 2014 median hospital wide cost of sepsis was reported at $32,421 ($USD 2014; IQR $20,745-$40,835) per patient [4], it is estimated that these initial inpatient costs only represent a third of the total cost burden with the other two-thirds attributed to lost productivity and other indirect medical costs [5,6]. Interventions to manage sepsis need to be clinically effective and cost-effective. A recent systematic review considered the cost-effectiveness of a range of sepsis interventions which ranged from dominant to dominated when compared to usual care, with early goal directed therapy, which focuses on the first six-hours of intervention, varying from dominant to $80,852 per life saved ($USD 2018) [7].

The current study is focussed on Victoria, Australia. In 2016/17 there were 33,220 hospital admissions (28,872 patients) with one or more of the sepsis diagnoses codes and 3,258 of these patients died during their episode of care, giving an “in-hospital” case fatality ratio of 11.2% [8]. Specific to Victoria, two studies have considered the economic, clinical and epidemiologic characteristics of patients with sepsis admitted to hospitals [9,10]. Sundararajan and colleagues reported the incidence of sepsis and severe sepsis, utilization of intensive care unit (ICU) resources, and hospital mortality with an overall hospital incidence of sepsis at 1.1%, with a mortality of 18.4% [9]. Of the septic patients, 23.8% received some care in an ICU and hospital mortality was 28.9%. Thursky and colleagues evaluated implementation of the Sepsis Pathway (key to the ‘Think sepsis. Act fast.’ Initiative [11]), which focused on six key actions within the first 60 minutes of sepsis recognition, across one hospital [10]. It was reported that compared to usual care, the 212 participants who received care based on a sepsis Pathway had significantly reduced time to antibiotics (55 minutes versus 110 minutes), as well as a significantly lower rate of ICU admission (17.1% versus 35.5%), length of stay (7.5 days versus 9.9 days) and sepsis related mortality 5.0% versus 16.2%) [10]. Hospital admission costs were also reported as a cost saving of $AUD 8,363 per patient on the Sepsis Pathway [10].

We report the economic evaluation of a state-wide scaling initiative that implemented the ‘Think sepsis. Act fast.’ Sepsis Pathway [11] across 10 of Victoria’s public health services (23 hospitals), which serviced 63% of the State’s population, or 15% of the Australian population. Based on this state and national representation, it is expected that the findings will have significant implications for future health policy and provision of health services. This evaluation had two research questions. Was the implementation of a state-wide Sepsis Pathway cost-effective in reducing mortality and/or hospital admission costs? What was the cost of implementing a state-wide Sepsis Pathway over 12 months?

## Methods

**Fig 1:**
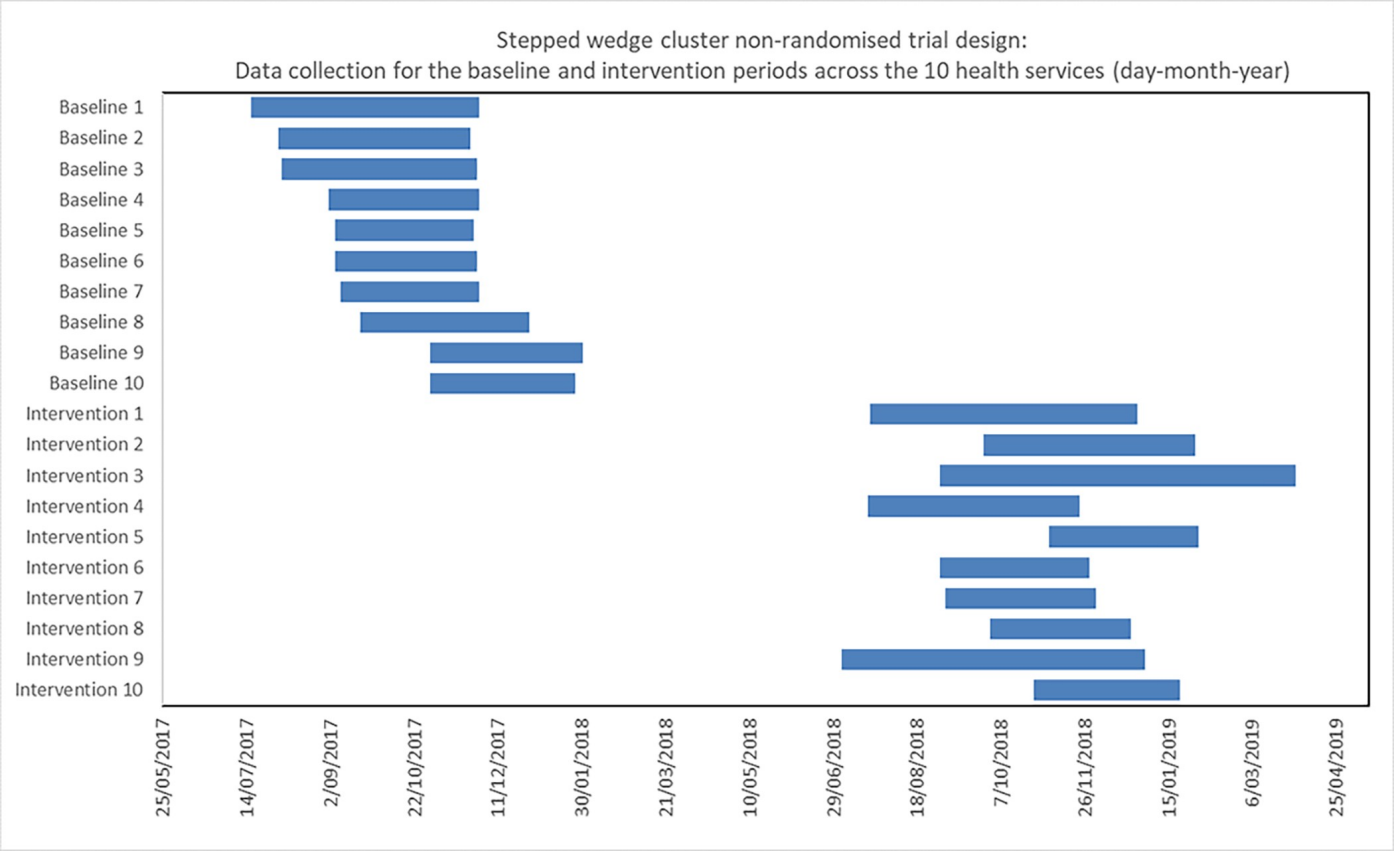
Stepped wedge cluster non-randomised trial design: Periods of data collection across the 10 health services.

This mortality-based cost-effectiveness analysis examined the impact of implementing an existing Sepsis Pathway (“Think sepsis. Act fast” [11]) across 10 of Victoria’s public health services. Individual health service Human Research Ethics Committee approval was required to participate in this economic evaluation with overarching ethics approval via the Melbourne Health Human Research Ethics Committee (QA2014075). A non-randomised stepped wedge cluster implementation study design [12] was used to implement the Sepsis Pathway. All 10 participating health services commenced with sepsis usual care (baseline data collection) and crossed over to the Sepsis Pathway (intervention data collection) at a self-determined time (Fig 1). The intervention data were collected prospectively, and the baseline data were collected retrospectively from the previous year to approximating the same seasonality of the intervention data collection period.

This project had three phases and three groups. The baseline phase involved the collection of health service data pre Sepsis Pathway implementation (Baseline group; Baseline Phase). The pilot phase (Pilot Group; Pilot Phase) of the Sepsis Pathway captured data while the pathway was being introduced in 1 or 2 areas of the hospital, most commonly the Emergency Department and/or an acute ward, to understand the barriers and facilitators to implementation that were specific to the health service. The pilot phase often identified barriers and facilitators, and utilised real time feedback to clinicians for on-pathway and off pathway sepsis episodes. Following the pilot phase, the Sepsis Pathway implementation plan was modified based on feedback from the pilot phase. It was then implemented at a whole-of-hospital level and this was reported as the intervention data (Intervention Group; Intervention Phase). While baseline, pilot and intervention data were collected, the current analyses only compare the baseline data to the intervention data.

### The intervention

The intervention included the Sepsis Pathway in addition to an implementation toolkit. In summary, the clinical pathway was designed to be a whole of hospital pathway that supported nurse initiation, the use of early warning criteria in combination with severity indicators. It was a medical record document, that included all elements of recognition, resuscitation and management including empiric antimicrobial therapy guidelines and a focus on the first 6 hours. Most hospitals utilised a paper pathway, and three of the hospitals adapted the pathway to their electronic medical record. The toolkit also included a guide to implementing a Sepsis Pathway, behaviour change strategies as well as quality improvement tools and templates, and engagement of consumers. A detailed description of the Think sepsis. Act fast. Sepsis Pathway, in addition to the implementation toolkit, is accessible from the Safer Care Victoria (SCV) website (Think sepsis. Act fast. [11]).

The following definitions were used to recognise suspected sepsis: (a) suspected or known infection plus two or more systemic inflammatory response syndrome (SIRS) criteria, and/or (b) two of more signs of severe sepsis (hypotension <100mmHg, altered mental state, and / or lactate greater than 2mmol/L). Sepsis was defined as suspected or known infection plus two or more SIRS criteria; severe sepsis was defined as the same as the sepsis definition with additional evidence of hypoperfusion (e.g. systolic blood pressure, elevated lactate); septic shock was defined as requiring inotropes and/or pressors to maintain blood pressure.

### The implementation

The ‘Think sepsis. Act fast.’ scaling collaboration (the collaboration) was a 12-month collaborative model of learning. The collaboration was established by Safer Care Victoria (SCV) and funded by the Better Care Victoria (BCV) Innovation Fund. Experienced clinicians and staff who had undertaken a sepsis improvement initiative was critical to the scaling approach and a partnership was formed with Royal Melbourne Hospital as the champion site to engage the 10 participating health services. The aim of the collaboration was to scale the sepsis pathway to 10 additional health services to improve outcomes for patients with sepsis. Each health service implemented a sepsis clinical pathway that was originally developed and implemented at Peter MacCallum Cancer Hospital and adopted by Melbourne Health in 2016–17 [10].

Melbourne Health was assigned to be the champion site responsible for developing capability and guiding implementation efforts. A clinical lead (KT) and project lead (KS) were recruited to oversee the change management process, address local barriers and risks, monitor project milestones, and assist in developing resources. Health services received support in the form of site visits, face-to-face meetings, one-on-one coaching, telephone, and electronic correspondence. Additionally, a database manager at 0.2 FTE was allocated for six months to manage the central REDCap database [13,14].

The collaboration was governed by a steering committee comprising a range of experts from administrative, clinical and government positions, as well as a consumer. SCV provided project governance and leadership support and worked closely with the Melbourne Health champions.

Hospitals were required to submit an expression of interest and to demonstrate interest in driving sepsis improvement with a high level of organisational readiness, clinical engagement and executive support. Each health service was funded to recruit a project officer (0.8 FTE) and a clinical lead (0.1–0.2 FTE) for 12 months at each service. These roles were expected to deliver the project in its entirety and act as sepsis champions in their hospital. Consumer engagement was a key requirement for all sites. Health services were permitted to adapt the pathway document to include local empiric antimicrobial guidelines, and formatting to meet local branding requirements.

Six face-to-face workshops were organised throughout the project to support project teams, build capability, and share learnings. The content of workshops varied, including health service updates, discussions, and lectures aimed at building capability based on upcoming project milestones. Key workshop topics included: introduction to the project, clinical considerations, lessons from the Melbourne Health experience, improvement science and approach to scaling, implementation, project and health economic evaluation, consumer engagement (two workshops), data collection, testing, piloting and sustainability.

Site visits by SCV project staff and Melbourne Health project leads were made to provide individualised support and coaching. An online platform called Basecamp [15] was used as a project management and team communication tool to allow a central place for sharing resources and knowledge among health services. The tool served as the storage platform of documents, which enabled a ‘one stop shop’ for all services.

The primary objectives of the program were to decrease the rate of inpatient sepsis-related mortality, decrease hospital mean and median LOS for sepsis-related presentations and decrease the rate of sepsis-related ICU admissions.

### Implementation evaluation

The intervention was compared to usual care sepsis management prior to the introduction of the intervention. The intervention was the implementation of the Hospital-wide Sepsis Pathway across all wards and emergency departments, and the elements of the Sepsis Pathway include six key actions within 60 minutes of sepsis recognition, 1) Oxygen administration, 2) Two sets of blood cultures, 3) Venous blood lactate, 4) Fluid resuscitation, 5) Intravenous antibiotics within 60 minutes of recognition, and 6) Monitoring observations and fluid balance.

Adherence to the Sepsis Pathway was via a medical record audit. Absence of documentation inferred non-adherence. There were two methods for reporting adherence, the first was a binary outcome (Yes / No) to report if the patient commenced on the Sepsis Pathway. The second was adherence to three of the Sepsis Pathway actions that were considered to be most feasible process measures: two sets of blood cultures prior to antibiotics, venous blood lactate and intravenous antibiotics within 60 minutes. The remaining three actions (oxygen administration, fluid resuscitation and monitoring observations and fluid balance) were more difficult to quantify and consistently capture as process measures.

### Population and setting

Participant consent was waived by the ethics committees as only deidentified “usual care” data were collected for adults admitted to hospital with a Sepsis diagnosis. All patient sepsis episodes were included in adults (>18 years) with a confirmed diagnosis of sepsis at one of the participating health services. There were no additional exclusion criteria. Sepsis-3 elements (hypotension, altered mental status, and elevated lactate) were included to identify patients with severe sepsis at risk of higher mortality [16]. The setting included 10 of Victoria’s public health services (the Collaboration) across 23 hospitals. One additional health service that participated did not obtain ethics approval to submit data to the collaboration. With respect to potential bias from the non-randomised clusters, the project Steering Committee was not aware of any key sepsis clinical staff who worked across multiple participating health services.

### Data and definitions

Retrospective baseline data collection was from July 2017 to January 2018 and prospective intervention data collection was from July 2018 to March 2019 (Fig 1). Cases were captured through a list of ICD-10 sepsis codes identified through the Victorian Admitted Episodes Dataset (VAED) supplied by the Victorian Agency for Health Information. Prospective pilot and implementation phase episodes were identified hospital wide through the VAED coding list extracted from the Victorian Agency for Health Information, in addition to active surveillance. Data were collected to determine location of sepsis identification, sepsis severity based on the collaboration criteria, clinical information about vital signs, pathology, antibiotic treatment, suspected or known infection diagnosis at presentation, final diagnosis at discharge, and patient outcomes such as LOS, ICU admission, readmission, and death.

The minimum recommended sample size for small hospitals (<100 beds) was 30 and large hospitals (>100 beds) was 100. A data collection form and data collection guide were developed and provided to each service to standardise the process. The data collection form included two pages of mandatory fields and two pages of optional fields. The focus of this evaluation compares baseline and implementation cohorts.

As a Collaboration, there were expected differences between the metropolitan and rural and regional health services. Potential differences included the time horizon (total days of data collection), the time of year, the rate of sepsis identification, as well as the method for sepsis identification based on local hospital systems. While these factors could not be modified due to the complexity of the collaboration, multiple regression analyses, which demonstrated that these factors did not influence the clinical outcome results, were completed and reported in a separate Think sepsis. Act fast. Evaluation report (Think sepsis. Act fast. [11]).

### The economic evaluation

This economic evaluation has been prepared with reference to the Consolidated Health Economic Evaluation Reporting Standards (CHEERS) Checklist [17]. All costs are presented in Australian dollars (AUD) 2018/19. The primary economic evaluation took a healthcare sector perspective, and the secondary evaluation took an expanded healthcare sector perspective which included the Victorian State Government costs of Sepsis Pathway implementation.

This economic evaluation examined both the *cost* and *effect* of implementing the Sepsis Pathway. The cost included health service expenditure for patients with an episode of care linked to sepsis as well as costs borne by the Victorian State Government’s Department of Health and Human Services through the BCV Innovation Fund which provided governance and project support for implementation of the scaling collaborative. The primary clinical *effect* included mortality and the primary process evaluation *effect* was adherence to the Sepsis Pathway in routine care. The choice of these outcome measures was influenced by the 2016/17 BCV funded pilot study at Melbourne Health (Victoria, Australia) which reported that the introduction of a Sepsis Pathway (effect = adherence to the key elements) improved mortality (effect = mortality) and reduced length of stay (component of cost) and intensive care unit ICU admissions (component of cost) [10].

### Time horizon

The study had a total time horizon of 12 months. Health services were advised to collect between 3–4 months of baseline data, 2–3 months of pilot data and 3–6 months of intervention data based on what was feasible for the individual health service (Fig 1). Due to the 12 month time horizon, no discount rate was applied to costs or consequences.

### Outcome

The primary outcome was in-hospital all-cause mortality, due to high rates associated with sepsis hospitalisations [2]. A reduction in in-hospital mortality rate was considered a valid measurement of effectiveness in the current study due to the synthesised evidence demonstrating that effective sepsis interventions can reduce mortality [18].

### Health service utilisation

Health service utilisation was based on individual patient data collection from the health service administration database onto a paper-based data collection tool which was then entered into the REDCap [13,14] database for analysis. Emergency department, acute and ICU admissions were measured via an admission time stamp and a discharge time stamp with the difference used to calculate health service utilisation reported as the length of stay (LOS) in days. Emergency department utilisation was also measured as a binary Yes/No outcome.

### Health service cost

As it is appropriate to use LOS as a proxy for cost within an economic evaluation investigating the implementation of a Sepsis Pathway [10], this costing methodology has been applied. As such, the acute hospital costs have been modelled from the Independent Hospital Pricing Authority data on National hospital costs from the Round 21 Financial Year 2016/17 report [19] with the 2016/17 cost per day ($1,972) inflated by consumer price index (CPI) [20] to represent a 2018/19 NPV cost per day in $AUD. The ICU hospital costs have been modelled from NSW Cost of Care Standards 2009/10 [21] with the 2009/10 cost per day in ICU ($4,028) inflated by CPI [20] to represent a 2018/19 NPV cost per day in $AUD. The emergency department costs have been modelled from the Independent Hospital Pricing Authority data on National hospital costs from the Round 20 Financial Year 2015/16 report [22] with the 2015/16 cost per emergency department presentation ($517 for an emergency department presentation without a subsequent acute admission and $977 with a subsequent acute admission) multiplied by CPI [20] to represent a 2018/19 NPV for cost per emergency department presentation in $AUD (Table 1).

**Table 1:** Cost data—units of measurement and unit costs for health care utilisation.

Health service costs, $AUD 2018/19	Unit description / data source	Unit	Cost per unit
**Emergency department presentation WITHOUT a subsequent acute admission**	Cost of an emergency department presentation WITHOUT a subsequent acute admission [17]	1 presentation	$543
**Emergency department presentation WITH a subsequent acute admission**	Cost of an emergency department presentation WITH a subsequent acute admission [17]	1 presentation	$1,027
**Acute admission**	Cost of an acute hospital admission per diem [14]	1 day	$2,052
**Intensive care unit** **admission**	Cost of an intensive care unit admission per diem [16]	1 day	$4,933

Local health service implementation costs were not included in the healthcare sector perspective as a substantial portion of this cost was borne by the Victorian State Government (i.e., the project manager and the clinical lead), in addition it was decided a priori that the individual health services were not required to report on all resources that contributed to local implementation, for example education sessions and awareness campaigns.

### Cost of implementation

The expanded healthcare sector perspective, which includes the government costs of Sepsis Pathway implementation, refers to the costs borne by the -BCV Innovation Fund within the Victorian State Government’s Department of Health and Human Services. BCV Innovation Fund resource utilisation was based on three cost categories. The first category was the direct project costs at each of the 10 health services and this included the project manager (usually a practice development nurse or advanced practice pharmacist) and clinical lead (usually an emergency or infectious diseases physician) to support implementation of the project. The second category was the direct project costs for the lead site (Melbourne Health) and this included a project manager (1.0 EFT), clinical lead (0.1 EFT) and data manager (0.1 FTE). The third category was the BCV internal resources and this included the staff that provided eadership, governance and co-ordination of the project as well as the cost of staff travel, a consumer representative, workshops and the evaluation. BCV resource costs were calculated based on the approved project budget as well as via additional cost and resource data provided by BCV. Cost data included costs allocated within the 2017/18 and 2018/19 financial years. Cost data from 2017/18 was inflated by CPI [20] to represent a 2018/19 NPV. Cost allocation to each of the 10 health services was calculated based on an even distribution of total BCV Innovation Fund costs to each of the 10 health services. Cost data did not include the in-kind contribution by the health services, by non-remunerated members of the steering committee, or by the consumers.

### Cost -benefit analysis

A simple cost-benefit analysis was also completed based on the cost of implementation, cost-savings associated with the patients admitted during the intervention period as well as the cost-savings associated with each statistical life saved [23].

### Patient and public involvement

Consumer representatives were approached through the SCV consumer group. From the point of project conception, a consumer representative was involved in the project steering committee. Participating health services were also required to ensure consumer representation at the local level. As such, consumers had input into the project design, developing the project evaluation framework, reviewing the reports and establishing the dissemination plan.

### Sensitivity analysis

**Fig 2:**
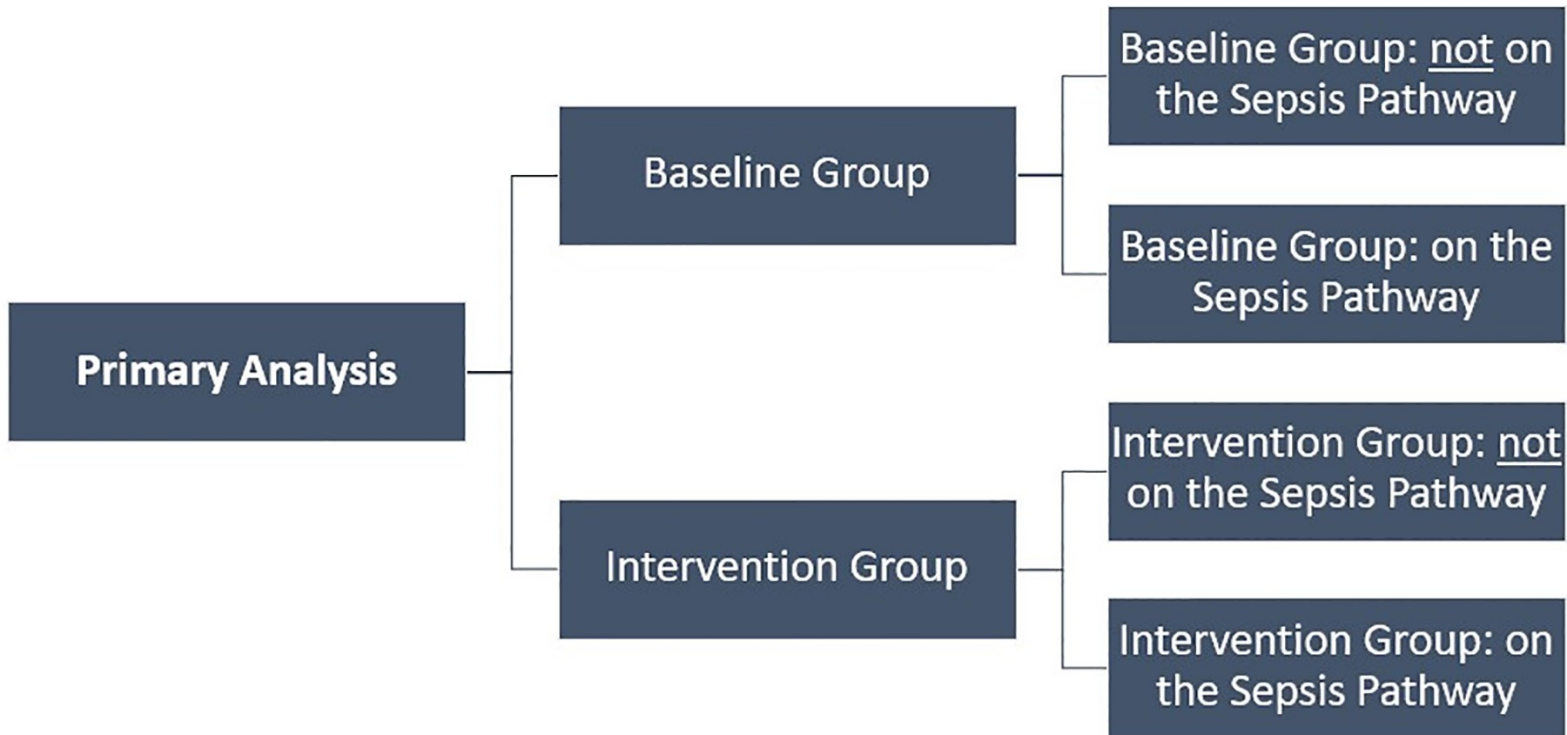
Patient selection for the primary analyses.

**Fig 3:**
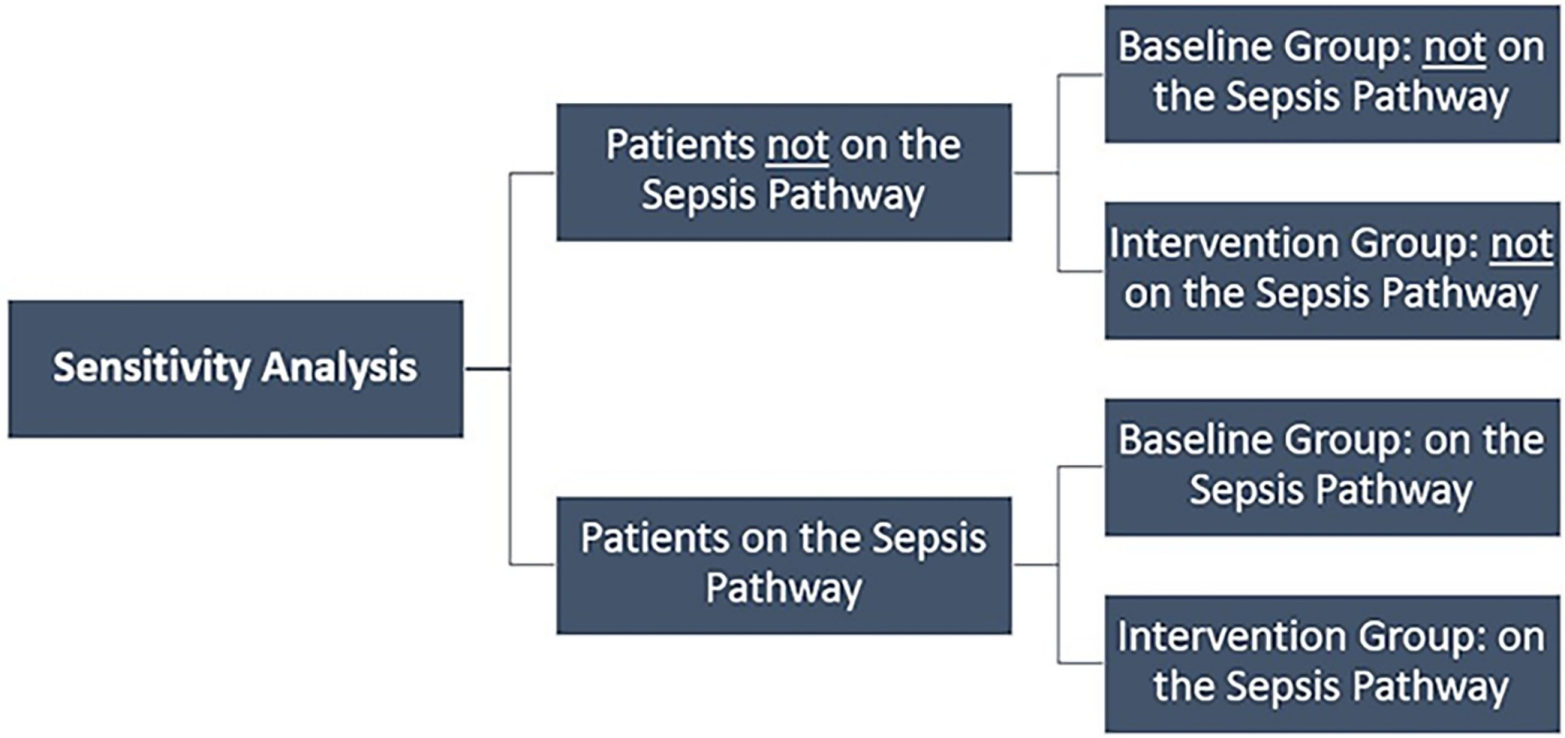
Patient selection for the sensitivity analyses.

This economic evaluation has also completed a sensitivity analysis to compare all patients (combined from baseline and intervention groups) on the Sepsis Pathway to all patients not on the Sepsis Pathway, in addition to the planned primary analyses comparing the baseline group to the intervention group. This was done to check the robustness of the primary economic evaluation and understand if it was impacted by the differences in both the process for data collection, and the rate of sepsis identification, between the baseline and the intervention groups (Figs 2 and 3).

### Statistical analysis and sample size calculation

As this was an evaluation of the scaling of an established effective intervention, sample size was determined pragmatically through pre-determined periods of data collection for the baseline, pilot and intervention phases, rather than via a sample size calculation. Across the 10 health services, the time horizon for data collection varied pending on the individual health service project implementation plan. It was expected that the baseline phase would be between 3 and 4 months, and that the intervention phase would be between 3 and 6 months.

Continuous parametric variables were analysed using a t-test and categorical variables were analysed using cross tabulation with Chi^2^ test to determine significance. The continuous parametric variables of length of stay and cost was also analysed using negative binomial regression, which accounted for hospital clustering, to check the robustness of the results due to the assumed skewed nature of this data. The mortality impact of the pathway was also assessed by estimating an adjusted intervention effect. This included adjusting the data for age, sepsis severity, and Charlson comorbidity index to estimate the lives saved. ICERs were determined using modelled individual patient cost and clinical outcome data with bootstrapping applied (5000 repetitions) to individual ICERS to determine the two-dimensional point estimate and confidence ellipses [24]. Stata, SPSS and Excel were used to complete the analyses. Significance was assumed at p<0.05.

## Results

There were 876 patients in the baseline phase over 987 days of data collection and 1,476 in the intervention phase over 1,244 days of data collection, with the days of data collection varying between the health services (Table 2; Fig 1). The average rate of sepsis identification per week was 7 patients at baseline, and 10 during the intervention, indicating a 38% increase in the rate of sepsis identification (which used an early warning criterion, but with no difference in proportion of sepsis severity). At baseline, 392 (44.7%) of patients were female and during the intervention 648 (43.9%) were female. The average age at baseline was 68.4 years (range 57.5 to 73.7 across the health services) and during the intervention was 66.8 (range 60.8 to 73.0). Table 3 details the demographic and clinician details of sepsis episodes, and the process measures during baseline and evaluation phases of the implementation, respectively.

**Table 2:** Participants included in the study.

	Baseline n = 876	Implementation n = 1,476
**Number (range across the 10 health services)**	876 (37–184)	1,476 (16–322)
**Days of data collection (range across the 10 health services)**	987 (83–136)	1,244 (84–212)
**Patients identified with Sepsis, per 30 days (range across the 10 health services)**	30.91 (11.28–40.59)	42.56 (5.39–60.38)
**Male n (%)**	484 (55.3)	828 (56.1)
**Age in years, mean (SD)**	68.4 (17.1)	66.8 (18.5)
Age adjusted Charlson Co-morbidity Index* (SD)	5.23 (2.46)	5.59 (2.50)
**Infection at presentation**		
**Suspected or known infection n (%)**	741 (84.6)	1,216 (82.4)
**Unknown source**	135 (15.4)	260 (17.6)
**Clinical criteria**		
**White cell count, mean (SD)**	13.8 (10.1)	13.4 (17.4)
**Neutrophil count, mean (SD)**	11.3 (9.0)	11.3 (26.0)
**Heart rate, mean (SD)**	104.7 (22.8)	105.4 (21.3)
**Respiratory rate, mean (SD)**	22.9 (6.0)	23.3 (6.4)
**Temperature in celsius, mean (SD)**	37.8 (1.6)	37.9 (1.5)
**Systolic blood pressure, mean (SD)**	122.4 (30.9)	126.2 (26.5)
**Altered mental state**	294 (33.6)	337 (22.8)
**Lactate mmol/L, mean (SD)**	2.8 (2.9)	2.3 (1.7)

**Table 3:** Clinical details of sepsis episodes, and process measures during baseline and evaluation phases of the implementation.

	Baseline n = 876	Implementation n = 1,476	
**Sepsis severity classification**			**Risk ratio (95% CI) p-value**
Sepsis (SIRS plus infection) n (%)	548 (62.6)	975 (69.3)	1.06 (0.99–1.12) p = 0.09
Severe sepsis n (%)	229 (26.1)	378 (26.6)	0.98 (0.85–1.12) p = 0.77
Septic shock n (%)	67 (7.6)	80 (5.4)	0.71 (0.52–0.97) p = 0.03
Criteria not met n (%)	32 (3.7)	43 (2.9)	0.80 (0.51–1.25) p = 0.32
**Location of sepsis**			
Patients managed in ED n (%)	808 (92.2)	1,360 (92.1)	
ED triage category			**Risk ratio (95% CI) p-value**
1 (seen immediately) n (%)	20 (2.5)	28 (2.1)	0.83 (0.47–1.47) p = 0.52
2 (seen within 10 mins) n (%)	320 (39.6)	691 (50.8)	1.28 (1.16–1.42) p<0.001
3 (seen within 30 mins) n (%)	391 (48.4)	550 (40.2)	0.84 (0.76–0.92) p<0.001
4 (seen within 60 mins) n (%)	74 (9.2)	91 (9.6)	0.73 (0.54–0.98) p = 0.04
**Final infection diagnosis at discharge**			
Clinically documented only n (%)	322 (36.8)	619 (41.9)	1.14 (1.02–1.26) p = 0.01
Microbiologically diagnosed infection (MDI) (blood stream) n (%)	284 (32.4)	305 (20.7)	0.64 (0.56–0.73) p<0.0001
MDI (non-blood stream) n (%)	216 (24.6)	356 (24.1)	0.98 (0.84–1.13) p = 0.77
Sepsis without focus n (%)	46 (5.3)	97 (6.6)	1.13 (0.80–1.60) p = 0.49
Sepsis excluded n (%)	8 (0.9)	99 (6.7)	7.33 (3.59–15.0) p<0.0001
**In patient mortality**			
Overall all-cause mortality n (%)	100 (11.4)	85 (5.8)	0.50 (0.38–0.66) p<0.0001
*Mortality by sepsis classification*			
SIRS plus infection n (%)	47 (8.6)	31 (3.2)	0.39 (0.25–0.61) p<0.0001
Severe sepsis n (%)	35 (15.3)	31 (8.2)	0.53 (0.33–0.85) p = 0.007
Septic shock n (%)	17 (25.4)	21 (26.3)	0.73 (0.38–1.38) p = 0.34
Sepsis criteria not met	1 (3.1)	2 (4.7)	1.2 (0.1–13.0) p = 0.88
**Length of stay (LOS)**			**Change, p-value**
LOS days, median (range)	5.6 (0–82)	4.2 (0–127)	-1.4 days, p<0.001*
LOS days, mean (SD)	9.1 (10.3)	6.2 (7.9)	-2.9 days
**Intensive care (ICU) admission**			**Risk ratio (95% CI) p-value**
Initial admission n (%)	201/855 (23.5)	210/1,358 (15.5)	0.66 (0.56–0.79) p<0.0001
Further ICU admissions during same episode n (%)	10/201 (4.9)	5/210 (2.4)	0.48 (0.17, 1.38) p = 0.16
ICU LOS days, mean (SD)	4.6 (6.2)	3.4 (2.8)	-1.2 days p = 0.02
Process measures during baseline and evaluation phases of the implementation			
**Sepsis managed on pathway**			**Risk ratio (95% CI); p-value**
All patients n (%)	43 (4.91)	1,151 (78)	15 (11.9–21.2) p<0.0001
Emergency department n (%)	43/808 (5.3)	1,059/1,360 (77.8)	14.6 (10.9–19.6) p<0.0001
Wards n (%)	0/68 (0)	92/116 (79.3)	p<0.0001
**Blood culture sets collected**			**Risk ratio (95% CI); p-value**
At least one set n (%)	754 (86.1)	1,386 (93)	1.09 (1.06–1.12) p<0.0001
Number of blood culture sets			
0 n (%)	122 (13.9)	90 (6.1)	0.4 (0.31–0.52) p<0.0001
1 n (%)	500 (66.3)	430 (31.0)	0.47 (0.43–0.51) p<0.0001
2 n (%)	225 (29.8)	910 (65.7)	2.20 (1.96–2.47) p<0.0001
3+ n (%)	29 (3.9)	46 (3.3)	0.86 (0.57–1.36) p = 0.53
**Lactate collected n (%)**	522 (59.6)	1,259 (85.3)	1.43 (1.24–1.52) p<0.0001
**Antibiotic timeliness (if not already on antibiotics)**			**Risk ratio (95% CI); p-value**
First dose systemic antibiotics given within 60 mins n (%)	270/722 (37.4)	710/1222 (58.1)	1.55 (1.40–1.73) p<0.0001
Minutes, median (range)	78.6 (0–1,780.0)	48.1 (0–751.0)	Change -38.8% p<0.0001
Minutes, mean (SD)	116.2 (138.9)	69.2 (70.0)	Change -40.4% p<0.0001
**Compliant with empiric sepsis guidelines n (%)**	501 (61.1)	1,076 (78.7)	Rate ratio 1.29 (1.21–1.37) p<0.0001
**Antibiotic Appropriateness** *	**Baseline**	**Implementation**	**% Change**
Adequate and Optimal	402/705 (57.0%)	847/1127 (75.2%)	+ 31.9
Inadequate and suboptimal	168/705 (23.8%)	187/1127 (16.6%)	- 30.3
Not assessable, not done or unknown	135/705 (19.2%)	93/1127 (8.3%)	- 56.8

*Not a mandatory field, collected in 80%.

### Adherence to the Sepsis Pathway

Adherence to the Sepsis Pathway was measured two ways. The first was a binary outcome (Yes / No) to report if the patient commenced on the Sepsis Pathway. The second was adherence to three of the Sepsis Pathway actions. There was a significant increase in the percentage of patients who commenced on the Sepsis Pathway from the baseline group (4.9%) to the intervention group (78.0%, p<0.00). There was also a significant increase in the percentage of patient who had two sets of blood cultures from the baseline group (42.9%) to the intervention group (70.9%, p<0.01), lactate completed from the baseline group (59.6%) to the intervention group (85.3%, p<0.01), as well as antibiotics within 60 minutes from the baseline group (36.2%) to the intervention group (55.1%, p<0.01).

### Mortality within the hospital admission

**Fig 4:**
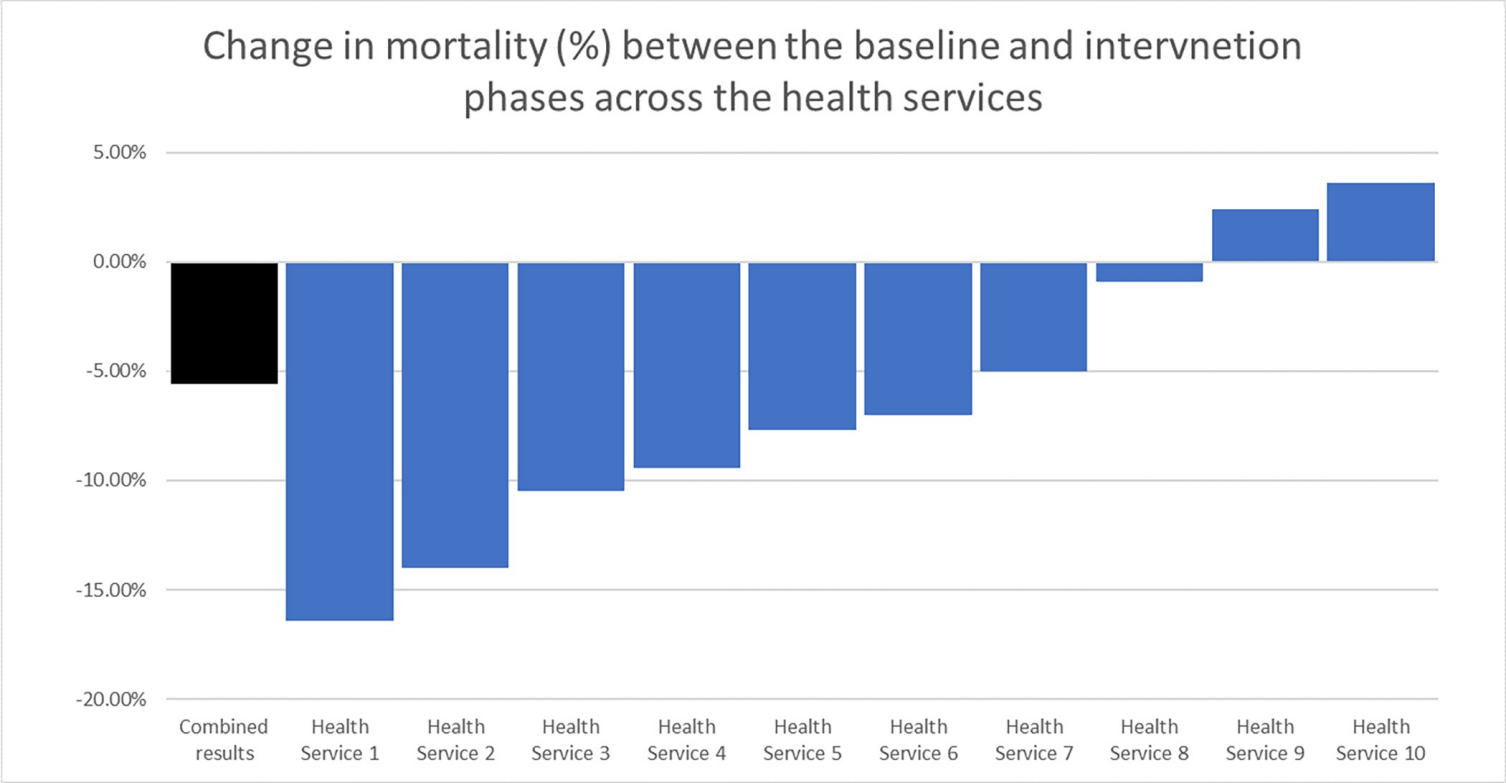
Change in mortality across the health services.

There was a significant decrease in the percentage of all-cause patient mortality from baseline (n = 100/876, 11.4%) to the intervention groups (n = 85/1,476, 5.8%, p<0.01) (Fig 4).

### Health service utilisation and cost

The number of patients with an episode of care linked to sepsis who had an emergency department presentation were consistent between the baseline (92.2%, n = 808) and the intervention groups (92.1%, n = 1,360). The emergency department length of stay had an observed reduction from the baseline group to the intervention group (mean difference (MD) -0.02 days 95%CI -0.04 to 0.00, p = 0.054). The acute admission length of stay, which had the intensive care unit length of stay removed, significantly reduced from the baseline group to the intervention group (MD -2.27 days 95%CI -3.00 to -1.57, p<0.00). The number of patients who had an intensive care unit admission decreased from 22.9% (n = 201) in the baseline group to 14.2% (n = 210) in the intervention group. The intensive care unit length of stay significantly reduced from the baseline group to the intervention group (MD -0.65 95%CI -0.86 to -0.43, p<0.00) (Table 4).

**Table 4:** Healthcare utilisation.

Emergency department count (%), length of stay in days (SD), and cost (SD)		Intensive care unit count (%), length of stay in days (SD), and cost (SD)		Acute admission count (%), length of stay in days (SD), and cost (SD)	
Baseline	Intervention	Baseline	Intervention	Baseline	Intervention
808 (92.2) 0.34 (SD0.24), $937 (SD281)	1,360 (92.1) 0.32 (SD0.22), $906 (SD298)	201 (22.9) 1.11 (SD3.71) $5,458 (SD18,281)	210 (14.2) 0.46 (SD1.53), $2,263 (SD7,525)	875 (100) 7.66 (SD 9.60) $15,712 (SD19,704)	1,476 (100) 5.38 (SD 7.72) $11,035 (SD15,844)

Mean length of stay per admission were 9.10 days (SD10.30) and $22,107 (SD $26,937.47) at baseline and 6.15 days (SD7.87) and $14,203 (SD $17,610) during implementation, significantly favouring the intervention group with 32.4% or a 2.9 day (95% CI 2.21 to 3.67) reduction in length of stay and a 35.8% or $7,904 (95% CI $5,907 to $9,901) reduction in cost. The effect size for LOS was 0.333 (95% CI 0.249 to 0.417) and for cost was (0.367 (05% CI 0.282 to 0.451). Using negative binomial regression, adjusting for age and the hospital clusters, the length of stay reduced by 32.2% (95% CI 21.8% to 41.2%), and the cost reduced by 35.7% (95% CI 27.6% to 43.0%).

The total cost for the baseline group was $19.4MIL to service 876 patients, compared to the total cost for the intervention group which was $21MIL (additional 8% in cost) to service 1,476 patients (additional 68% of patients). Extrapolated out, the 1,476 patients in the intervention group saved $11.7MIL and 4,280 bed days.

Across the health services the emergency department cost reduced from the baseline group to the intervention group (MD -$31; 95%CI -$56 to -$7, p = 0.01). While the results are statistically significant, due to the magnitude ($31) they may not be meaningful to health services as a useful cost saving initiative. In addition, the modelled emergency department costs were episodic, based on a subsequent acute admission or not, resulting in minimal variation and therefore a higher likelihood of a significant finding (Table 4).

The acute admission cost, which had the intensive care unit cost removed, significantly reduced from the baseline group to the intervention group (MD -$4,677; 95%CI -$6,131 to -$3,223, p<0.00). The intensive care unit cost also significantly reduced from the baseline group to the intervention group (MD -$3,196; 95%CI -$4,254 to -$2,138, p<0.00) (Table 4).

### Cost of implementation for the extended healthcare sector perspective

Cost of implementation was $1,845,230 across the 10 health services (Table 5). For the intervention group, when the implementation costs were evenly attributed, the cost per patient was $1,250. For the intervention group, when the per patient implementation cost ($1,250) was added to the per patient health service utilisation cost ($14,203), the combined cost was $15,453. When this combined cost for the intervention group is compared to the health service utilisation cost of the baseline group ($22,107), there was a cost savings of $6,654 (-$6,654; 95%CI -$8,458 to -$4,850, p<0.00) for the intervention group.

**Table 5:** Cost of implementation.

Element	Unit description / cost in base year^2^	Number of units	Total Cost	
			In base year	NPV 2018/19 FY
**Cost category 1: direct project costs at each of the 10 health services**				
Project Manager	NPV in 17/18 FY $85,000 x 10 sites (0.8 FTE per site or 8.0 FTE in total)	8.0 FTE	$850,000	$867,850
Clinical Lead	NPV in 17/18 FY $35,000 x 10 sites (0.1 FTE per site or 1.0 FTE in total)	1.1 FTE	$350,000	$357,350
Additional funds to support local project leads throughout the Hume region	NPV in 17/18 FY Additional Funds distributed across a rural health service for multiple project leads	Block funding	$118,182	$120,664
**Category 1 sub-total**				**$1,345,864**
**Cost category 2: direct project costs for the lead site**				
Clinical Lead	12 months	0.2 FTE	$63,636	$64,973
Data Manager	12 months	0.1 FTE	$13,636	$13,923
Project Manager	12 months	0.9 FTE	$109,091	$111,382
Project Manager	6 month extension	0.5 FTE	$54,545	$55,691
**Category 2 sub-total**				**$245,968**
**Cost category 3: SCV internal resources**				
Senior project officer	NPV in 18/19 FY $68,181 for 0.45 FTE. Multiplied over 18 months	0.75 FTE	$102,273	$102,273
Chair of the Steering Committee	NPV in 18/19 FY 0.045 FTE over 18 months (18 days)	0.05 FTE	$11,867	$11,867
Manager of Innovation Projects	NPV in 18/19 FY 0.2 FTE over 18 months (78 days)	0.2 FTE	$51,425	$51,425
SCV Travel costs in 2017/18	3 occasions of travel to rural and regional Victoria	Block funding	$376	$385
SCV Travel costs in 2018/19	4 occasions of travel to rural and regional Victoria	Block funding	$1,113	$1,113
Consumer representative	NPV in 17/18 FY Sessional payments at the Steering Committee meetings for attendance plus travel)	8 meetings	$2,909	$2,970
Workshop 1^1^	NPV in 17/18 FY Catering costs = $1,338	1 workshop	$1,338	$1,366
Workshop 5^1^	NPV in 18/19 FY Catering costs = $319	1 workshop	$319	$319
Economic evaluation	NPV in 17/18 FY = $80,000	One collaboration analysis / report and 10 health service analyses / reports	$80,000	$81,680
**Category 3 sub-total**				**$253,398**
**TOTAL NPV 2018/19**				**$1,845,230**

### Cost effectiveness outcome measures for patients with an episode of care linked to sepsis

The following figures are a graphical representation of the ICER value with the variation shown around the ICER (seen as a 50%, 75% and 95% confidence ellipses). To interpret the graph the first step is to recognise if the point estimate is above or below the horizontal line. This indicates if the incremental cost of the intervention group is more than (above the line) or less than (below the line) the incremental cost of the baseline group. The second step is to recognise if the point estimate is to the left or right of the vertical line. This indicates if the incremental effect (mortality) of the intervention group was greater than (to the right) or less than (to the left) the incremental effect of the baseline group. In the case of mortality, a reduction in the incremental effect is a better outcome and therefore the bottom left quadrant is the dominant quadrant as it represents lesser cost for a better outcome.

**Fig 5:**
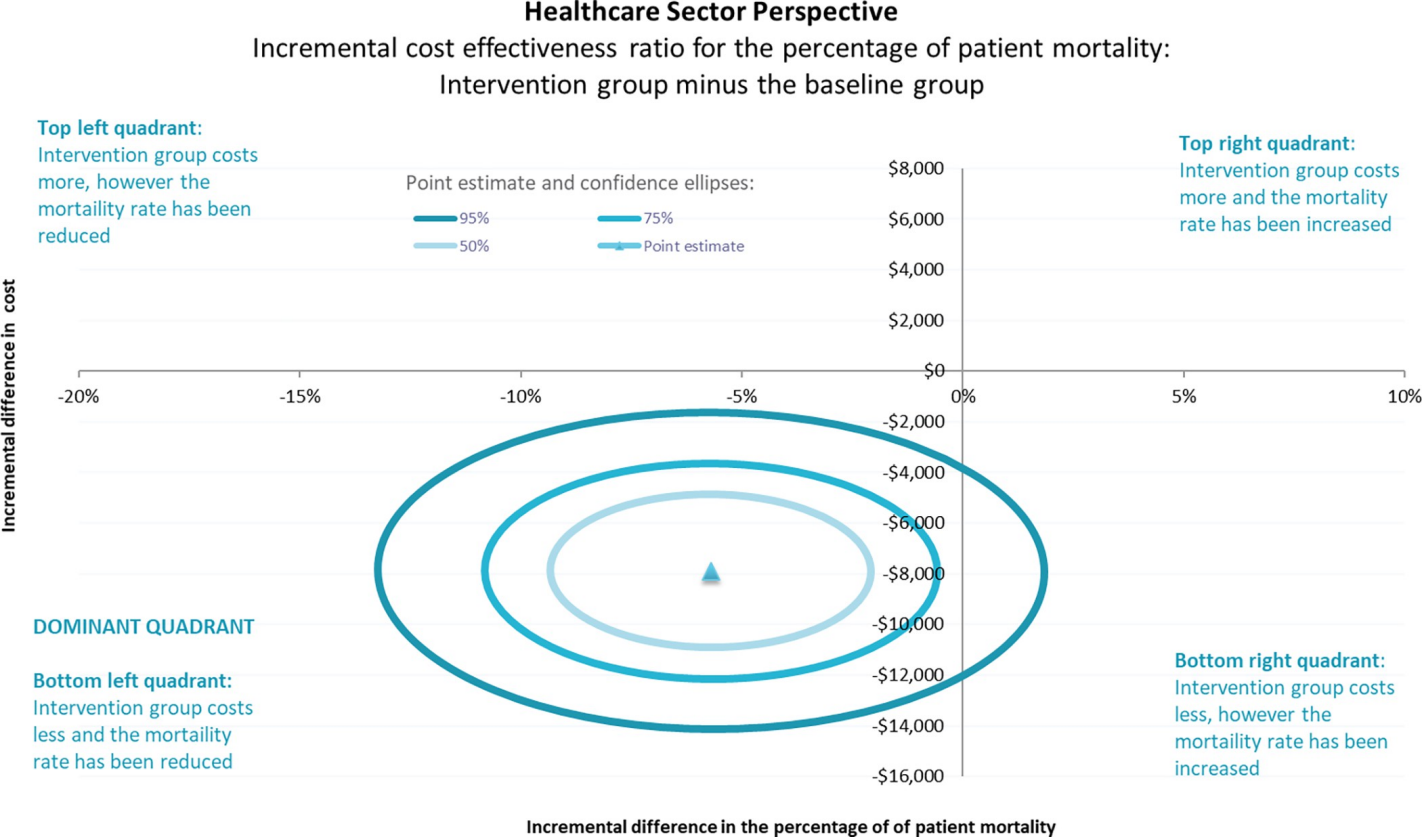
Incremental cost effectiveness ratio for patient mortality (percentage) from a healthcare perspective.

**Fig 6:**
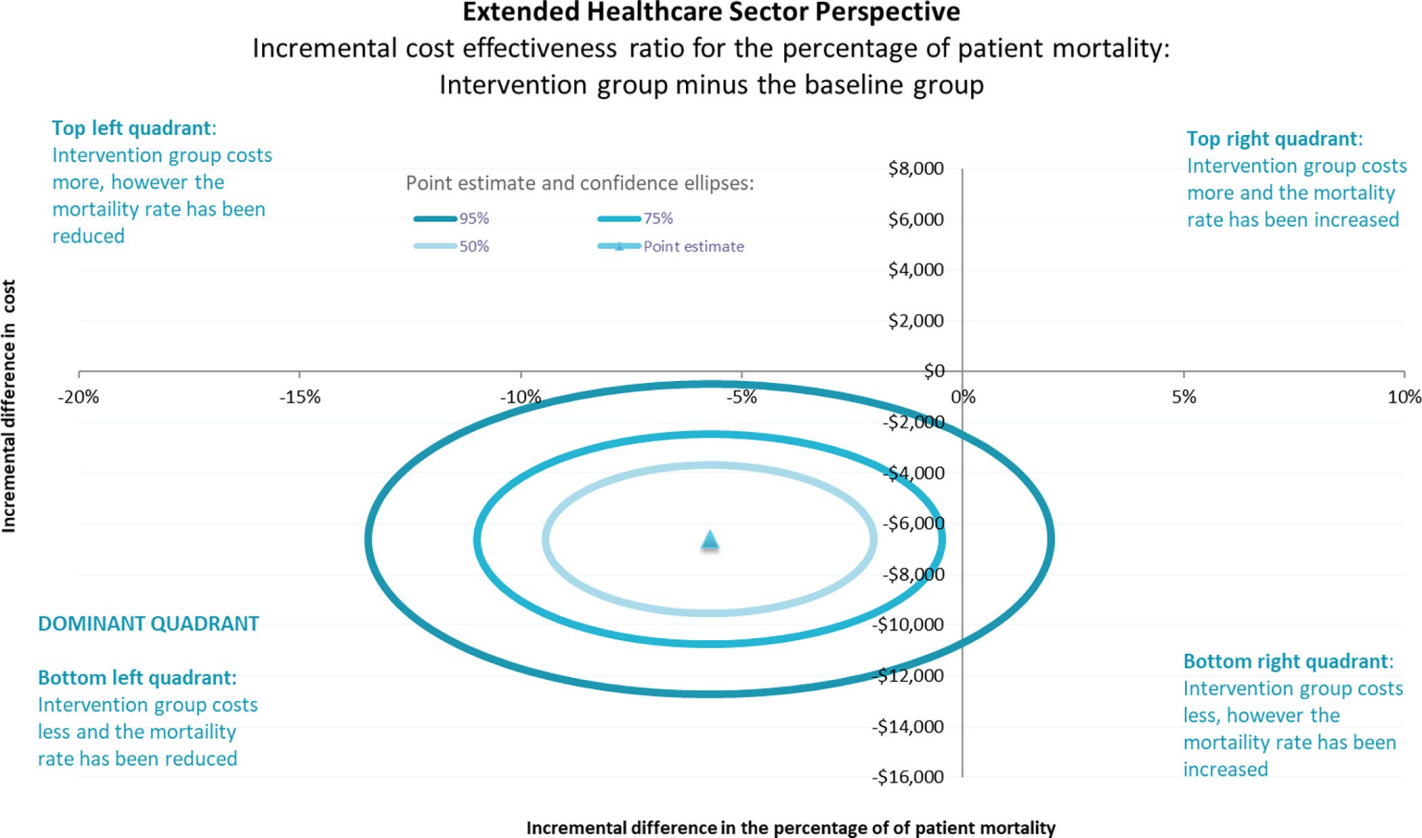
Incremental cost effectiveness ratio for the mortality (percentage) from an extended health care perspective (inclusive of implementation costs).

From a healthcare sector perspective (health service costs only), the results of the cost effectiveness analyses demonstrate that the implementation of the Sepsis Pathway was cost effective. That is, the implementation of the Sepsis Pathway was a dominant intervention compared to usual care as it resulted in better clinical outcomes at a lesser cost (Fig 5). From an extended healthcare sector perspective (health service costs combined with implementation costs), the results of the cost effectiveness analyses continue to demonstrate that the implementation of the Sepsis Pathway was cost effective (Fig 6). That is, the implementation of the Sepsis Pathway resulted in better clinical outcomes at a lesser cost despite the addition of the BCV implementation costs. In both scenarios, the 50% and 75% confidence ellipses were in the dominant quadrant, and the 95% confidence ellipse entered the bottom right quadrant indicating a small probability that the difference in cost was due to chance.

### Cost -benefit analysis

The cost-savings associated with each statistical life saved was based on previously published data which reported that when adjusting for age, sepsis severity and comorbidities (Charlson Comorbidity Index), it was estimated that the pathway saved 52 lives during the implementation period (see report; Think sepsis. Act fast. [11]). With each statistical life saved valued at $7,150,165 (AUD 2017 $7,016,060 multiple by CPI for 2018/19 value) [23], the value of 52 lives saved was $371,808,582.

Combining the cost of Sepsis Pathway implementation (-$1,845,230) with the cost-savings associated with the patients admitted during the intervention period (+$11,666,304) and the cost-savings of the 52 statistical lives saved (+$371,808,582), the net cost-benefit of the Sepsis Pathway Implementation Collaboration was $383MIL.

### Sensitivity analysis from the healthcare sector data

The following sensitivity analyses compared all patients (combined from baseline and intervention groups) on the Sepsis Pathway (n = 1,194) to those not on the Sepsis Pathway (n = 1,158). This sensitivity analyses were consistent with the original healthcare sector analyses and have provided evidence that the differences in the identification rate of Sepsis and the process for data collection, between the baseline and the intervention groups, did not influence these results.

In reference to adherence and mortality, there was a significant increase in the percentage of patients who had two sets of blood cultures from the non Sepsis Pathway patients (49.7%) to the Sepsis Pathway patients (70.9%, p<0.01), lactate completed from the non Sepsis Pathway patients (60.4%) to the Sepsis Pathway patients (90.6%, p<0.00), as well as antibiotics within 60 minutes from the non Sepsis Pathway patients (36.5%) to the Sepsis Pathway patients (59.3%, p<0.00). There was a significant reduction in the percentage of patient mortality from the non Sepsis Pathway patients (11.3%, n = 128/1,132) to the Sepsis Pathway patients (5.3%, n = 57/1,081, p<0.00).

In reference to healthcare utilisation, the emergency department length of stay significantly reduced from the non Sepsis Pathway group to the Sepsis Pathway group (MD -0.02; days 95%CI -0.04 to -0.002, p = 0.03). The acute admission length of stay, which had the intensive care unit length of stay removed, significantly reduced from the non Sepsis Pathway group to the Sepsis Pathway group (MD -2.43; days 95%CI -3.11 to -1.75, p<0.00). The intensive care unit length of stay significantly reduced from the non Sepsis Pathway group to the Sepsis Pathway group (MD -0.59; 95%CI -0.80 to -0.38, p<0.00).

In reference to healthcare cost, the emergency department cost significantly reduced from the non Sepsis Pathway group to the Sepsis Pathway group (MD -$31; 95%CI -$59 to -$12, p<0.00). The acute admission cost, which had the intensive care unit cost removed, significantly reduced from the baseline group to the intervention group (MD -$4,988; 95%CI -$6,391 to -$3,585, p<0.00). The intensive care unit cost also significantly reduced from the baseline group to the intervention group (MD -$2,919; 95%CI -$3,943 to -$1,895, p<0.00).

In combination, the non Sepsis Pathway patients had an average total length of stay of 8.8 days (SD 10.7) with an average cost of $21,180 (SD $26,661) per patient, compared to the Sepsis Pathway patients with an average total length of stay of 5.7 days (SD 6.6) with an average cost of $13,327 (SD14,966). This represents a significant 3.0 day reduction in total length of stay (-3.0; 95%CI -3.8 to -2.3, p<0.00) and a significant $7,943 reduction in cost (-$7,943; 95%CI -$9,684 to -$6,201, p<0.00). Extrapolated out, the 1,194 patients on the Sepsis Pathway saved $9.5MIL and 3,582 bed days.

## Discussion

The scaling implementation of a nurse led, whole of hospital pathway across 10 health services [22 hospitals) was a dominant intervention as both mortality and health service costs significantly reduced for patients who were managed with the Sepsis Pathway. The main drivers for cost reduction were the reduced ICU admissions and the reduction in overall hospital length of stay. The results demonstrated a saving of $11.7MIL and 4,280 bed days for the intervention group when compared to usual care, across the health services (with an average of 5 months of intervention data collection per health service). The return on investment represents significant value for money as the investment of $1.8MIL through the BCV Innovation Fund resulted in a return of $11.7MIL, representing a 6-fold return on investment over 5 months. Although sustainability was not measured in the current study, sustainability was measured during the projects pilot study with sustained Sepsis Pathway adherence demonstrated at 12 months [10]. Both the pilot study and the current study invested in similar sustainability strategies and these included medical staff education during orientation and regular nursing in-services [10].

A key strength of the current study is the inclusion of 10 of Victoria’s public health services from both metropolitan and rural and regional areas, which services 63% of the State’s population. This study was not designed as a clinical trial, it was rather designed as Phase T3 research (implementation and dissemination research [25]) to implement and scale an existing intervention. Despite the tight 12 month timeline, the scaling methodology was successful across hospitals of varied size and resources. Another strength is the inclusion of the sensitivity analysis which provided evidence that the differences in the identification rate of sepsis and the process for data collection, between the baseline and the intervention groups, did not influence these results. Methodological limitations include the non-randomised pre-post study design, the lack of individual patient-level cost data from each of the health services and the pragmatic approach which allowed a variable time horizon for the baseline and implementation phases at each health service, due to individualised implementation processes. While there was an increased rate of sepsis diagnoses, the proportion of severe sepsis remained consistent (26.1% at baseline and 26.6% post implementation) indicating that the lower mortality post implementation was not due to lesser severity sepsis patients being identified. However, it is possible that those patients who did not enter the sepsis pathway may have had atypical presentations of sepsis and therefore a higher mortality. Additionally, other changes over time independent of the initiative (eg general awareness of sepsis) may also have impacted on outcomes. As such, it is acknowledged that there are alternative explanations for the apparent treatment effect. To address this, multiple regression analyses were completed in the Think sepsis. Act fast. Evaluation report (Think sepsis. Act fast. [11]) and these demonstrated that the time horizon (total days of data collection), the time of year, the rate of sepsis identification, as well as the method for sepsis identification based on local hospital systems, did not influence the clinical outcome results. This enabled the research team to confidently state that the implementation of the Sepsis Pathway was a plausible explanation for the reduction in both mortality and health service costs.

In the current study, 92% of patients with an episode of care linked to sepsis were identified in the ED which was much greater than the findings of Sundararajan et al. who identified 70.8% of patients with Sepsis had an ED admission over a 4-year period in Australia [9]. Shorr et al., reported that ED costs were not substantially affected by implementation of a Sepsis protocol [26]. This was similar to the current study where the ED length of stay had an observed reduction from the baseline group to the intervention group (MD -0.02 days, p = 0.054), and the ED cost marginally reduced from baseline to intervention group (MD -$31, p = 0.01). With respect to ICU, the $3,196 (p<0.00) reduction in costs in the current study were consistent with the findings by Thursky et al., who also reported significant reduction in ICU costs between the historical and Sepsis Pathway cohorts of $5,470 (p<0.01) [10]. Most, but not all, previous studies which have also focussed on early Sepsis interventions reported an associated reduction in mortality [27–29].

Despite there being no change in the length of stay in the ED, the total length of stay, and subsequently total admission costs reduced, with a significant 2.9 day reduction in total length of stay (p<0.00) and a significant $7,904 reduction in cost (p<0.00). These results are consistent with the findings of Thursky et al., [10] which reported a significant difference in the admission cost per patient on the Sepsis Pathway between the historical and Sepsis Pathway groups of $8,363 (p = 0.048) and a 2.4 day reduction in overall length of stay (from 9.9 days to 7.5 days; p<0.05). Similarly, Shorr et al., reported that the implementation of a Sepsis protocol was associated with a median reduction in overall hospital length of stay by 5 days (p = 0.023) [26].

Given the results clearly demonstrate the implementation of the Sepsis Pathway is cost effective, meaning it has delivered both better clinical outcomes and reduced cost, exploration of further scaling programs is supported. Should the ‘Think sepsis. Act fast.’ Sepsis Scaling Collaboration scale further, it is recommended that there is ongoing diligence with the evaluation of cost effectiveness, to ensure the benefits continue to be realised. One of the major challenges in sepsis programs internationally is the lack of a gold standard to reliably identify patients with sepsis, in addition, diagnostic coding is notoriously challenging as it depends upon clinician documentation. It is possible that the clinician documentation was influenced by the sepsis awareness programs and therefore represents an important confounder in this study. It is possible that increased awareness of sepsis resulted in milder cases being recognised and thus overestimating the effect of the intervention. We were not able to adjust for all potential markers of severity, such as lactate or acute mental status, as data were missing for a significant minority of patients. However, the proportion of patients with severe sepsis and septic shock was similar before and after the intervention, suggesting that the intervention did impact on mortality, but the magnitude of the effect should be interpreted with caution. It should also be noted that the effect size does not impact on our conclusion that this intervention was dominant (that is, it reduced mortality and reduced cost). This program which was initially developed in 2013 prior to the 2016 Sepsis-3 criteria has adopted a modified combination criteria requiring the presence of suspected or known infection with 2 or more SIRS criteria and/or the presence of 2 or more severe criteria (hypotension, elevated lactate, and altered mental status). The outcomes of this scaling support the routine implementation of an early warning criteria (such as SIRS or NEWS). The consumer and clinical stakeholders were overwhelmingly supportive of the pathway and the qualitative evaluation can be accessed via the public report (Think sepsis. Act fast. [11]).

### Conclusion

This economic evaluation sought to identify how the outcomes of the ‘Think sepsis. Act fast.’ Sepsis Scaling Collaboration demonstrated value for money. The results of the cost effectiveness analyses consistently demonstrated that the implementation of the Sepsis Pathway was cost effective by delivering better clinical outcomes, specifically reduced mortality, at a lesser cost. The reduction in hospital length of stay and reduction in cost extrapolated across the 1,476 patients in the intervention group saved $11.7MIL and 4,280 bed days. Therefore, when considering the investment of $1.8MIL by the BCV Innovation Fund, there was a 6-fold ($11.7MIL) return on investment and more importantly, a significant improvement in clinical outcomes for patients across Victoria was delivered, demonstrating very good value for money. Based on the significant Victorian state representations, it is expected that the findings will have significant implications for future health policy and provision of health care services nationally and internationally.
